# The Hospital Management and Treatment of Infectious Diseases.—II
*A paper read before the Royal Sanitary Institute, Leeds.


**Published:** 1922-10

**Authors:** A. E. Pearson

**Affiliations:** (Medical Superintendent, Seacroft Infectious Diseases Hospital)


### THE HOSPITAL MANAGEMENT AND TREATMENT OF INFECTIOUS DISEASES.*—II.

By A. E. PEARSON, L.R.C.P., M.R.C.S. (Medical Superintendent, Seacroft Infectious Diseases Hospital).

### On Diagnosis.

A CORRECT diagnosis in infectious diseases is not always an easy matter, and a considerable number of mistakes are made. It is often a difficult matter to place these patients so as to ensure safety to themselves and to other patients in the hospital. The comparatively frequent occurrence of these cases accentuates my plea for more single-room isolation. Though there are known to be simple bacteriological tests which could be applied before admission of the patient, these are not often resorted to. There is too great a tendency to rely on one clinical sign in arriving at a diagnosis—for instance, a scarlet rash for scarlet fever, throat exudations for diphtheria, diarrhœa and dry tongue for typhoid, and so on. Concomitant signs and symptoms being disregarded, a wrong diagnosis is fairly often arrived at.

A word or two about bacteriological tests. These may lead one astray occasionally. In diphtheria, for instance, a negative bacteriological test does not necessarily negative a diagnosis of diphtheria. One should take into account the many non-acid-producing, non-pathogenic pseudo-baccilli occasionally found, and there also seems to be a tendency to come to a bacteriological diagnosis on morphological appearances alone. For instance, take the bacillus auris found so often in the ear discharges of scarlet fever, the xerosis bacillus and so on. Again, I do not regard a negative agglutination blood test for typhoid conclusive proof of absent typhoid. This is a test of resistance and I have fairly often found agglutination absent in very severe typhoids, quite positive by clinical signs. Direct blood cultures in septicæmia are, in my opinion, the surest bacteriological test. In scarlet fever a diagnostic sign, advanced by Dr. Meredith Young, in an article in the "Practitioner," might be used, *i.e.*, the production of petechial patches by pinching the skin, after producing dilatation of venous vessels of the limb by a bandage.

Measles and scarlet, measles and diphtheria, measles and smallpox, measles and typhus, chicken pox and smallpox, and others having points of similarity, have often been mistaken for one another and have given rise to much anxiety to the administrative mind.

### Incidence of Complications, Treatment and Mortality.

Scarlet fever admissions, which have averaged about 1,000 cases annually for a number of years, have not shown a marked increase or decrease, except during the years 1916, 1917 and 1918, when the average fell to 588, the cause of the decrease not being apparent.

The death-rate of scarlet fever, which was 5·6 per cent. in the first quinquennial period of my time in the fever hospitals (1891 to 1895), has since fallen gradually to 1·6 per cent. in the last quinquennial period, 1916 to 1920; the death-rate for 1920 was 0·8 per cent., and for the current year up to date, 0·8 per cent. for 1210 patients whose treatment was completed. On these figures, it would appear that some 50 to 100 patients escape death every five years, as compared with the earlier periods, or about 15 fewer deaths each year. But it has been stated to me that, though the death-rate is much lower, damage to the constitution left by the disease may be greater. I do not find this to be the case, as the following figures show. Complications, and consequent damage temporary and permanent, have fallen correspondingly. I have taken rheumatism, heart murmurs, otitis media, rhinitis and albuminuria as expressions of sepsis, virulence and damage :—

| | 1892-1912 (20 yrs.) | 1920 | Nov. to Jan. 1921-22. |
|---|---|---|---|
| Rheumatism ... | 2·5 | 2·1 | 3·3 |
| Endocarditis ... | 1·3 | 1·7 | 1 |
| Otitis media ... | 11·4 | 6·5 | 6 |
| Mastoid disease ... | 0·7 | 0·2 | 0·6 |
| Rhinitis ... ... | 9·3 | 9 | 7·3 |
| Albuminuria ... | 9·1 | 4·3 | 3·9 |
| Uræmia ... ... | 0·2 | 0·1 | Nil. |
| Meningitis ... | 0·4 | Nil. | Nil. |
| Death-rate ... | 3·1 | 0·9 | 0·6 |
| Relapse-rate ... | 2 | 2·5 | 2·4 |

Against the popular view, true relapses do occur in scarlet fever, either from auto-infection or from segregation with other cases, especially during the period of maximum prevalence of the disease. I think one may regard albuminuria as a qualitative expression of toxæmia in all bacterial diseases. And, again, in comparison with earlier years, we used to have three or four severely hypertoxic malignant cases every year with early fatal results; these are now very rarely seen.

As in the seasonal incidence of numbers of cases, so in the incidence of complications in scarlet fever, we should, I think, closely follow meteorological conditions. Following the annual increased prevalence in October, November and December, we find that the incidence and severity of complications and the tendency to fatal results increases gradually to February. After this month, from March, a steady decrease in these takes place to August. In my view, apart from reasons of segregation of persons in the winter months, absence of sunshine, increased humidity, especially observed after a very hot summer and gradually *increasing low* temperatures (mean temperatures and earth temperatures) by decreasing body resistance, are important factors in determining incidence and severity of not only scarlet fever, but all septic diseases, as, say, erysipelas, puerperal fever and septic cases of diphtheria. An extraordinary wave of sepsis seems to be present during the present month (February) in the increase of complications.

*A paper read before the Royal Sanitary Institute, Leeds.

Taking 242 scarlet cases now in hospital—

| | | | | |
|---|---|---|---|---|
| Albuminuria ... | from 4·3 per cent. | in 1920 | to | 5 per cent. |
| Rheumatism ... | „ 2·1 ,, | „ ,, | „ | 4·5 „ |
| Otitis media ... | „ 6·5 ,, | ,, „ | „ | 10·4 „ |
| Rhinitis ... | „ 9 ,, | „ „ | „ | 11·2 „ |
| " Return " case rate | „ 2·5 ,, | „ ,, | „ | 8 „ |

Relapses have been also more than ordinarily frequent, and this percentage is likely to be increased before discharge of the patients.

The chief cause of death in scarlet fever is a secondary septicæmic fever following on the primary specific toxic fever, which occurs in patients mostly under five years of age. In treatment, by the use of a mixed streptococcic vaccine adopted by me during the last three years, I am fairly certain that this secondary blood-infection has been largely prevented, a mitigated recovery usually ensuing, whereas formerly many of this class of patient died on or about the fourteenth day of disease. The ideal treatment of scarlet fever, I think, when it can be put into practical working, will be vaccination by autogenous vaccines prepared from individual patients. It is most difficult to control septic and other suppurative complications by local antiseptics.

The admission of diphtheria cases shows an increase, though the actual increase seems to me to be more apparent than real, for, as I have stated before, many of these patients, having patched throats, are assumed, without having had bacteriological endorsement, to be diphtheria. Fairly often, the diagnosis arrived at in this way is incorrect. During the first five years of my hospital residence there were fifteen cases of diphtheria admitted; during the last five years there were 2,940. I well remember when diphtheria was practically non-existent in Leeds. Then, about the year 1906, I remember the disease breaking out in one class of scholars at St. Stephen's School at Kirkstall. Thereafter it spread quickly to all parts of Kirkstall, afterwards to Chapeltown, and since it has seemed to be more or less present in every part of Leeds. The question arises in my mind, could this advancement of incidence have been stopped, had we had at hand immunisation by such a method as toxin—antitoxin as now practised in America and suggested by Dr. Copeman's recent report on diphtheria? The answer, I think, tends towards the affirmative.

In treatment we depend on antitoxin. The success of this depends, in my opinion, not so much on the huge doses sometimes necessary and now largely advocated, as upon the time which has elapsed since the start of the illness and its use. In this connection Schick's test for toxin content might well be used to determine dosage, and so probably and possibly tend to prevent the use of extravagantly large doses. I need not remark that antitoxin is expensive. Our annual bill at the hospital, on this account alone, is some £500 to £600.

The death-rate, thanks to the antitoxin treatment brought out by Behring and Kitesato in the year 1890, has fallen from 20 per cent. in 1890 to 6·5 per cent. in the year 1920, in the Leeds hospitals.

In typhoid fever, cases admitted have fallen from an annual average of 170 during the years 1890 to 1910 to an average of 72 during the quinquennial period 1911 to 1915, and to an average of 28 during the past five years ended 1920. Of 17 cases under treatment during the year 1921 there was not one death. Complications in the disease are now fewer than formerly; it is now about three years since a perforated bowel occurred. Partly, no doubt, due to decline in numbers treated and virulence, I think the complication list may have been reduced also by vaccine treatment now practised, which also, I believe, tends to prevent relapses and " carrier " incidence In the treatment of prolonged and serious types of this disease, as in all septicæmias in which the blood has been denuded of complement, blood transfusion, if obtainable, ought, I think, to be considered.

### " Carrier " Cases: " Return " Cases.

The last point, but by no means the least important, is the " carrier " patient who, after apparent recovery from an infectious disease, and after a longer or shorter period, causes an illness of the same kind to others. In typhoid and diphtheria we have bacteriological tests which give fairly reliable information as to absence of the specific micro-organism. In scarlet fever, however, we have no such reliable means of enabling us to make dogmatic statements of freedom from infection. We can only be guided by naked-eye appearances of throat, nose, ears and skin. The occurrence of the " return " scarlet case is disappointing and annoying alike to the hospital management and to the patient's friends. The number is about two to three per cent. of those treated. I may briefly endeavour to indicate the probable causes which seem to operate in all fever hospitals. We cannot eliminate the primary case as a cause which is due apparently mainly to the occurrence of septic catarrhal conditions. These and " return " cases seem more likely to arise at the termination of the annual scarlet fever outbreak—*i.e.*, January to March—when meteorological conditions favour their incidence by reducing resistance on the return home of the primary patient to houses sometimes overcrowded with susceptibles; the house probably not always being in a perfect sanitary condition. Personally I do not think that desquamation of the skin can be seriously considered as a contributory cause. My own view is that the cause frequently lies in infected clothes, owing to defective disinfection, either at the hospital or at the patient's home, or to articles which have escaped disinfection. The clothes are subjected to steam in a disinfector. I believe that this operation is not always effective. The operation requires great care. I have known macroscopic organisms to survive the usual operation by exposure of verminous clothes to steam in one of these machines. A somewhat striking feature of the connection of clothes in septic diseases like scarlet fever, diphtheria and erysipelas is that of 559 persons over 12 years of age admitted in 1921 engaged in business who were attacked by one of these diseases, 194 were engaged in working on clothes or cloth, or 34·7 per cent., tailors and tailoresses coming first with 15·2 per cent. There were only two laundresses in that number.

CONCLUSION.

In conclusion, fever hospitals exist at a large cost to the community, first, to promote recovery from infectious diseases; and, secondly, to prevent the spread of, and if possible to eradicate, infectious diseases. Do fever hospitals fulfil these objects? After a long and somewhat eventful experience, I think they tend to do so. In the earlier part of my time smallpox and typhus were prevalent, though intermittently, to a considerable extent; perhaps we may expect a recurrence of smallpox under present vaccination conditions, though for some years in Leeds these two diseases have been practically non-existent. Typhoid cases are admitted in fewer numbers each year. Scarlet fever and diphtheria show no marked decline in numbers. This, I think, is partly due to the working of the Infectious Diseases (Notification) Act since its adoption, and to some extent a consequent increase of cases having a doubtful or wrong diagnosis; also partly due to improved methods of diagnosis. But in these latter diseases the complication rate, the type and the mortality have markedly decreased. May not these decreased rates indicate a possible future diminution of case incidence, and possibly eventual total extinction?

